# Development and validation of a machine learning prediction model for postoperative arrhythmia after left atrial appendage occlusion

**DOI:** 10.1097/MD.0000000000050556

**Published:** 2026-09-11

**Authors:** Liang Xu, Siquan Niu, Shaohua Yang, Yan Wang, Menglu Liu, Xutan Qin, Yujie Zhao

**Affiliations:** a Department of Cardiology, Zhengzhou Seventh People’s Hospital, Zhengzhou, Henan, China; b Key Medical Laboratory of Arrhythmia, Zhengzhou, Henan, China.

**Keywords:** arrhythmia, left atrial appendage occlusion, machine learning, Predictive model, Web-based risk calculator

## Abstract

This study aimed to develop an effective predictive model for postoperative arrhythmia following left atrial appendage occlusion using a machine learning (ML) algorithm. The objective was to identify risk factors of postoperative arrhythmia and provide accurate predictions for patients undergoing left atrial appendage occlusion (LAAO). Patients who underwent LAAO at the Seventh People’s Hospital of Zhengzhou City between June 2016 and June 2018 were included. A comprehensive set of preoperative data, including demographic characteristics, medical history, preoperative scores, and surgical details, was collected. A simulated annealing feature selection algorithm was applied to identify the most relevant variables. Eight ML algorithms were evaluated using receiver operating characteristic- area under the curve analysis and clinical decision curve analysis. The SHapley Additive exPlanations algorithm was used to interpret the predictions of the optimal model. A total of 322 patients were included in the study. Among the 8 ML algorithms, eXtreme Gradient Boosting demonstrated an overall favorable performance, with a receiver operating characteristic - area under the curve of 0.7153 (95% CI: 0.6498–0.7808) in the testing set. The model also exhibited good calibration, with a Brier score of 0.114. Decision curve analysis indicated that the model provided net clinical benefit, with the highest net benefit observed within a threshold range of 0% to 25%. Key predictive variables included occluder size, gender, height, weight, and a history of hypertension. This study developed an eXtreme Gradient Boosting-based model that effectively predicts postoperative arrhythmia following LAAO. Further validation across larger, diverse cohorts is needed to optimize its generalizability and clinical applicability.

## 1. Introduction

Atrial fibrillation (AF) occurs in 1% of adults and more than 10% of heart surgery patients.^[1]^ The global prevalence of AF is estimated to be approximately 46.3 million individuals. By 2050, it is projected that 16 million people in the United States will have AF.^[2]^ Moreover, AF is associated with a substantial risk of stroke, which increases with age.^[3]^ This risk is largely attributable to thrombus formation. Previous studies have shown that left atrial thrombus occurs in approximately 13% to 17% of patients with both non-valvular and valvular AF, with 57% to 90% of thrombi located in or originating from the left atrial appendage (LAA).^[4]^

To prevent stroke in patients with AF, left atrial appendage occlusion (LAAO) has emerged as an important preventive strategy.^[5]^ However, LAAO may further predispose patients to postoperative arrhythmia. Several studies have reported that LAAO is associated with an increased risk of early postoperative atrial fibrillation (POAF), with an odds ratio of 3.88.^[6]^ Although the postoperative risks associated with LAAO have led to ongoing debate regarding its necessity as a stroke prevention strategy, the number of procedures performed worldwide continues to increase. A study from Germany reported that the number of endocardial occlusion procedures increased from 5259 to 5917 over 5 years,^[7]^ while in the United States, the number rose from 6069 to 29,083 during the same time frame.^[8]^ Given that postoperative arrhythmia following LAAO may further contribute to increased mortality risk and healthcare burden,^[9]^ early identification and prediction of postoperative arrhythmia risk in patients undergoing LAAO is of critical importance.

The application of machine learning (ML) in the healthcare industry is gradually becoming more prevalent.^[10]^ ML is well-suited to address problems that are challenging to resolve with traditional statistical methods. It excels in classification and regression tasks.^[11]^ It is possible that ML may be more effective than logistic regression in predicting postoperative complications due to its relative freedom from the limitations of traditional statistical analysis.^[12]^

A number of studies have employed ML models to predict the occurrence of POAF in patients presenting with postoperative arrhythmia. Lu Yufan and colleagues constructed a gradient boosting and support vector machine regression model to predict the outcome of POAF.^[10]^ Pablo Antúnez-Muiños and colleagues developed multivariate analysis methods and ML algorithms for the purpose of predicting device-associated thrombotic outcomes.^[13]^ Nevertheless, our research revealed a paucity of studies investigating the prediction of postoperative arrhythmia after LAAO.

The objective of this study was to develop ML models to predict the risk of postoperative AF development using general demographic characteristics and indicators of underlying disease conditions and the perioperative period.

## 2. Method

### 2.1. Patients

This study was initiated in January 2017. We retrospectively enrolled patients who underwent LAAO at the Department of Thoracic Surgery, Zhengzhou Seventh People’s Hospital, between June 2016 and June 2018. Data curation, model development, and validation were completed by December 2023. Prior to data collection, the study protocol was reviewed and approved by the Institutional Review Board of Zhengzhou Seventh People’s Hospital (approval number: IEC-C-007-A07-V1.0).

### 2.2. Criteria for patient inclusion

1. Age ≥18 years.
2. Symptomatic non-valvular AF.
3. Documented failure of at least 1 antiarrhythmic drug, with established indications for radiofrequency catheter ablation for AF.
4. CHA2DS2-VASc score ≥2 (≥ 3 in women) and at least one of the following criteria:
  a. Unsuitable for long-term anticoagulation treatment;
  b. Thromboembolic events still occurring despite long-term standardized anticoagulation therapy;
  c. HAS-BLED score ≥3.
5. Coronary computed tomographic angiography (CCTA) confirmed that the maximal diameter of the LAA ostium, measured at all ostial angles, was ≤30 mm.
6. Transthoracic echocardiography (TTE) demonstrated a maximal anteroposterior left atrial diameter < 50 mm. Additionally, the LAA must be clearly visualized. The predicted effective working depth of the LAA must be at least 70% of the maximal ostial diameter. A repeat preoperative TTE is required to confirm that the maximal anteroposterior left atrial diameter remains ≤50 mm.
7. Written informed consent signed voluntarily by the patients or their authorized legal representative.

### 2.3. Criteria for patient exclusion

The exclusion criteria were designed to minimize confounding factors that could independently influence arrhythmia occurrence, and to ensure that postoperative arrhythmia was primarily related to LAAO-associated clinical and anatomical characteristics:

1. Patients with congenital heart disease, valvular heart disease, unstable angina pectoris, acute myocardial infarction within the past 3 months, acute stage of myocarditis, cardiomyopathy, cor pulmonale, severe neurological disorders (e.g., severe disturbance of consciousness, severe neuroinfections), mental disorders (including substance abuse, drug dependence, and conditions precluding effective patient cooperation), medically or surgically uncontrolled thyroid dysfunction, autoimmune diseases, hematological disorders, malignancies, or severe systemic infections.
2. Concurrent diagnosis of other bradyarrhythmia (e.g., atrioventricular block, sick sinus syndrome) or malignant ventricular arrhythmia (e.g., sustained ventricular tachycardia).
3. Repeated percutaneous coronary intervention performed within ≤ 12 months after initial coronary arteriography or percutaneous coronary intervention; or presence of ≥ 95% stenosis in one or more major coronary arteries (i.e., left anterior descending artery, circumflex artery, right coronary artery).
4. Presence of New York Heart Association functional class IV heart failure despite standardized optimal medical therapy; or left ventricular ejection fraction ≤35% as assessed by TTE.
5. Presence of left atrial or LAA thrombus confirmed by transesophageal echocardiography or CCTA.
6. Presence of abnormal liver function (aspartate aminotransferase [AST] or alanine aminotransferase >3 × upper limit of normal) or impaired renal function (serum creatinine > 3.5 mg/dL or creatinine clearance <30 mL/min).
7. Patients with a history of cardiac surgery within the past 6 months.
8. Patients with contraindications to radiofrequency catheter ablation, including current pregnancy, known hypersensitivity to contrast agents, and other contraindications.
9. Patients with estimated life expectancy <12 months.
10. Inability to complete CCTA or digital subtraction angiography examinations due to contraindications (e.g., claustrophobia).

## 3. Data collection

### 3.1. Definition of postoperative arrhythmia

Postoperative arrhythmia was defined as any newly documented or recurrent clinically significant arrhythmic event occurring within 2 months after the LAAO procedure. Given the retrospective design, arrhythmia events were identified based on documented diagnoses in the electronic medical records, which were established by attending cardiologists.

The diagnosis of arrhythmia was supported by 12-lead electrocardiogram (ECG) and/or ambulatory ECG monitoring performed during routine postoperative follow-up. Ambulatory ECG monitoring was generally conducted for 24 hours or longer, according to standard clinical practice.

Arrhythmic events were considered valid if they met at least one of the following criteria:

1. Arrhythmia documented on a 12-lead ECG;
2. Arrhythmia documented on ambulatory ECG monitoring with a duration of ≥30 seconds;
3. A clear arrhythmia diagnosis recorded in the medical record requiring clinical attention or treatment adjustment.

Patients with transient arrhythmia who meet the above criteria were included in the analysis. For AF, no predefined AF burden cutoff was applied, as the study endpoint was based on clinically diagnosed arrhythmic events rather than continuous rhythm surveillance.

### 3.2. Data baseline and correlation analysis

A baseline was established for comparison between patients in the arrhythmia and non-arrhythmia groups. The 2 groups were then compared with respect to their respective differences using the chi-square test and *t* test.

### 3.3. Missing data management

As this was a retrospective study based on routinely collected clinical data, missing values were unavoidable. Variables with excessive missingness (>20%) were excluded from model development. For the remaining variables, missing continuous data were imputed using the median value, and missing categorical data were imputed using the most frequent category.

All imputation procedures were performed using the training set only, and the same imputation parameters were subsequently applied to the testing set to avoid information leakage.

### 3.4. Class imbalance management

The study population exhibited an imbalance with postoperative arrhythmia occurring in a minority of patients. To account for this, stratified ten-fold cross validation (CV) was applied during model training to preserve the class distribution within each fold. Model performance was evaluated using metrics that are robust to class imbalance, including receiver operating characteristic - area under the curve (ROC-AUC), sensitivity, and specificity.

## 4. Model construction

### 4.1. Division of test and training sets

The data set comprising observations from June 2016 to September 2017 was employed as the training set, while the data set comprising observations from October 2017 to June 2018 was employed as the testing set.

### 4.2. Variable screening

A simulated annealing (SA) feature algorithm is employed for the purpose of variable selection. The algorithm’s initial point of departure is based on the analogy between the annealing process of solids in physics and general combinatorial optimization problems. The algorithm is employed to identify the most pertinent features from the training set, thereby contributing to the prediction of postoperative arrhythmia.

Compared with traditional regression-based variable selection methods such as Least Absolute Shrinkage and Selection Operator, the SA algorithm does not rely on linearity assumptions and is more suitable for identifying optimal feature subsets in complex, nonlinear clinical datasets. Therefore, SA was adopted as a model-agnostic feature selection strategy in this study.

### 4.3. Prediction models

In this study, we have chosen 8 different ML algorithms: decision trees, random forests, eXtreme Gradient Boosting (XGBoost), Light Gradient Boosting Machine, elastic networks, k-nearest neighbours (KNN), support vector machines (SVM), and multilayer perceptron. These algorithms were chosen to fully consider the performance of different types of models on the dataset. In order to achieve the optimal performance of the model, we adopt a Bayesian hyperparameter optimization approach, which enables the model to identify the optimal parameters. A ten-fold CV method was used on the training set.

### 4.4. Model validation and selection

In this study, we initially evaluated the performance of the model in the test set based on the ROC-AUC, which is a crucial performance metric that enables us to identify the model that performs the best in distinguishing whether or not a postoperative arrhythmia occurs. Following the selection of the optimal models, we proceeded to utilize decision curve analysis (DCA) to assess the clinical utility of the selected models. DCA is employed to ascertain the practical value of the selected models in real-world clinical settings.

### 4.5. Model visualization and interpretation

Model performance and interpretability were evaluated using learning curves and SHapley Additive exPlanations (SHAP) values, respectively. The learning curve demonstrates the efficacy of the model under varying training set sizes, whereas the SHAP values elucidate the influence of each feature on the model’s predictions, thereby facilitating an understanding of the underlying decision-making process.

### 4.6. Statistical analysis and software

All statistical analyses and ML model development were performed using the R software (version 4.3.2). Continuous variables were assessed for normality prior to analysis. Variables with approximately normal distributions were expressed as mean and compared using the *t* test. Variables with skewed distributions were expressed as median and compared using the Wilcoxon rank-sum test. Categorical variables were presented as counts and percentages and compared using the chi-square test or Fisher’s exact test, as appropriate. A two-sided *P*-value < .05 was considered a statistically significant difference.

## 5. Results

### 5.1. Data baseline analysis

A total of 322 patients were included in this study, of whom 45 developed postoperative arrhythmia. Baseline clinical characteristics of patients with and without postoperative arrhythmia are summarized in Table 1.

**Table 1:** Demographic and baseline characteristics of patients with and without postoperative arrhythmia

Characteristic	Arrhythmia		
	No, N = 277*	Yes, N = 45*	*P*-value†
Age, yr	67 (59, 73)	71 (64, 74)	.098
Height, meters	1.68 (1.60, 1.73)	1.60 (1.56, 1.69)	.002
Weight, kg	71 ± 13	68 ± 12	.084
BMI	25.9 (23.2, 28.0)	26.0 (23.7, 27.7)	.961
Creatinine, μmol/L	65 (54, 78)	64 (54, 77)	.743
Urea, mmol/L	5.64 (4.62, 7.30)	6.11 (5.00, 7.54)	.315
ALT, U/L	23 (16, 38)	22 (15, 29)	.371
AST, U/L	24 (19, 33)	24 (19, 31)	.558
Hematocrit	0.43 (0.39, 0.46)	0.39 (0.37, 0.43)	<.001
Hemoglobin, g/L	136 ± 20	127 ± 18	.003
Platelet, ×10^9^/L	189 (152, 225)	181 (149, 221)	.640
LA diameter, mm	40.6 ± 6.1	42.6 ± 6.3	.051
LVEF, %	58 (53, 63)	57 (53, 62)	.876
Procedural time, min	245 (200, 305)	220 (195, 280)	.199
Occluder size, mm	27.0 (24.0, 30.0)	30.0 (27.0, 33.0)	<.001
CHA2DS2-VASc score	4.00 (3.00, 5.00)	4.00 (3.00, 5.00)	.667
HAS-BLED score	2.00 (2.00, 3.00)	2.00 (2.00, 3.00)	.757
Gender			<.001
Female	110 (39.7%)	32 (71.1%)	
Male	167 (60.3%)	13 (28.9%)	
Classification of AF			.878
Persistent	132 (47.7%)	22 (48.9%)	
Paroxysmal	145 (52.3%)	23 (51.1%)	
BNP			.715
Normal	109 (39.4%)	19 (42.2%)	
Abnormal	168 (60.6%)	26 (57.8%)	
Warfarin			.227
No history of drug use	268 (96.8%)	42 (93.3%)	
History of drug use	9 (3.2%)	3 (6.7%)	
NOAC			.393
No history of drug use	48 (17.3%)	11 (24.4%)	
History of drug use	229 (82.7%)	34 (75.6%)	
Antiplatelet			.435
No history of drug use	200 (72.2%)	35 (77.8%)	
History of drug use	77 (27.8%)	10 (22.2%)	
Hypertension			.518
No	109 (39.4%)	20 (44.4%)	
Yes	168 (60.6%)	25 (55.6%)	
Diabetes mellitus			.720
No	210 (75.8%)	33 (73.3%)	
Yes	67 (24.2%)	12 (26.7%)	
Coronary heart disease			.636
No	162 (58.5%)	28 (62.2%)	
Yes	115 (41.5%)	17 (37.8%)	
Congestive heart failure			.518
No	174 (62.8%)	26 (57.8%)	
Yes	103 (37.2%)	19 (42.2%)	
Stroke			.575
No	202 (72.9%)	31 (68.9%)	
Yes	75 (27.1%)	14 (31.1%)	
Bleeding history			.365
No	270 (97.5%)	43 (95.6%)	
Yes	7 (2.5%)	2 (4.4%)	
LAA morphology			.870
Chicken-wing	81 (29.2%)	11 (24.4%)	
Cactus	36 (13.0%)	7 (15.6%)	
Windsock	47 (17.0%)	9 (20.0%)	
Cauliflower	113 (40.8%)	18 (40.0%)	
Vasovagal reaction			>.999
No	275 (99.3%)	45 (100.0%)	
Yes	2 (0.7%)	0 (0.0%)	
Residual shunt			>.999
No	275 (99.3%)	45 (100.0%)	
Yes	2 (0.7%)	0 (0.0%)	
Pericardial effusion			.512
No	236 (85.2%)	40 (88.9%)	
Yes	41 (14.8%)	5 (11.1%)	
Cardiac tamponade			.599
No	270 (97.5%)	45 (100.0%)	
Yes	7 (2.5%)	0 (0.0%)	
Electrical cardioversion			.265
No	160 (57.8%)	22 (48.9%)	
Yes	117 (42.2%)	23 (51.1%)	

* Median (IQR); Mean ± SD; n (%).

† Wilcoxon rank-sum test; Welch two-sample t-test; Pearson’s Chi-squared test; Fisher’s exact test.

BMI = body mass index, ALT = alanine aminotransferase, AST = aspartate aminotransferase, LA = left atrium, LVEF = left ventricular ejection fraction, AF = atrial fibrillation, BNP = B-type natriuretic peptide, NOAC = Non-vitamin K antagonist oral anticoagulants, LAA = left atrial appendage.

### 5.2. Simulated anneal variable screening

**Figure 1.**
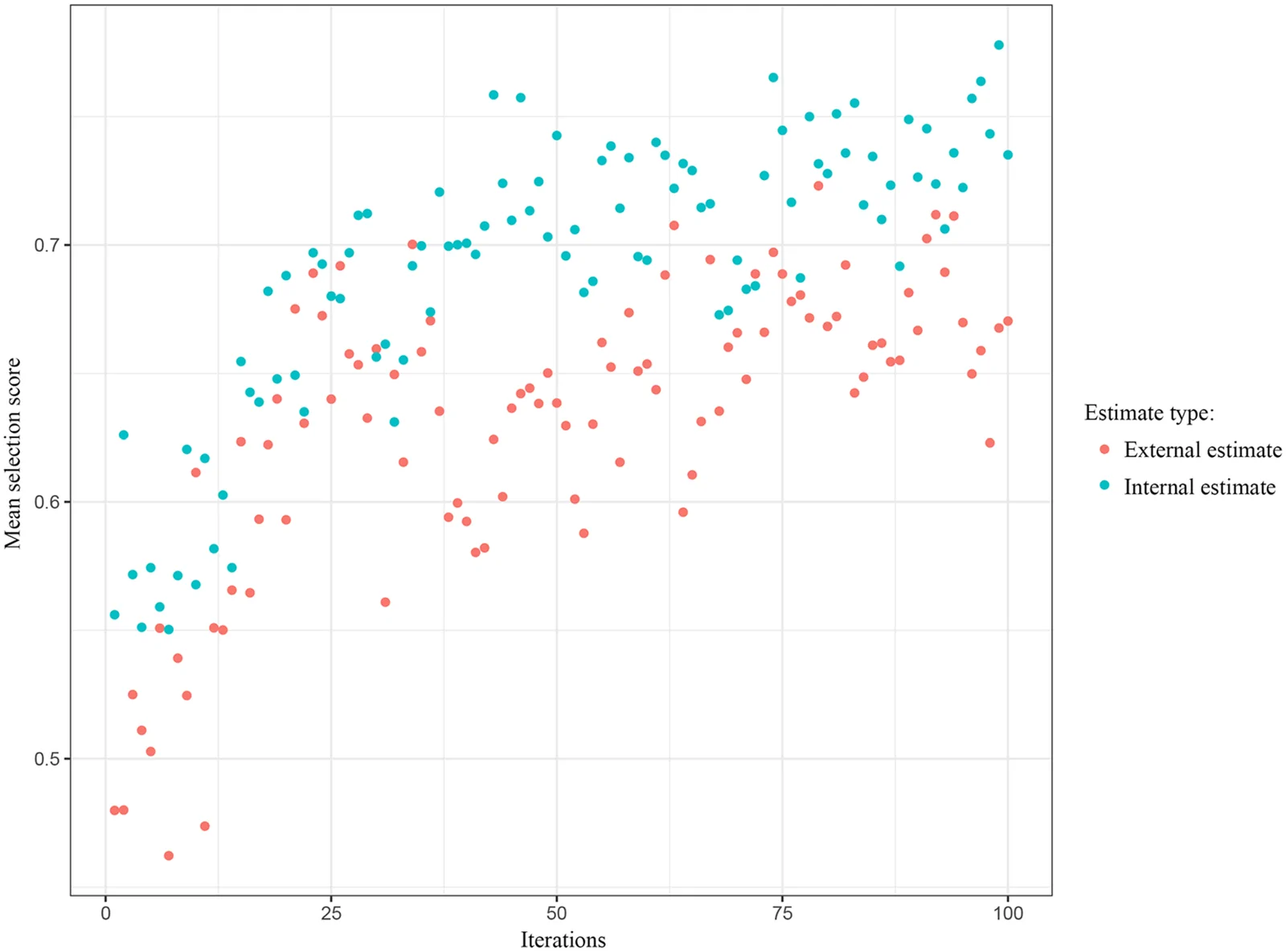
Feature selection process based on simulated annealing.

SA–based feature selection was applied to screen 35 candidate variables, with a maximum of 100 iterations. The optimal feature subset was determined based on external validation performance, with maximization of the external ROC as the primary criterion. During the iterative process, the external ROC increased and reached a stable maximum, after which inclusion of additional variables did not result in further performance improvement. At this optimal iteration (Fig. 1), a subset of 13 variables achieved the best balance between predictive performance and model complexity, whereas models with fewer variables showed reduced performance and models with more variables suggested redundancy. Consequently, 13 variables were retained for final model development, including occluder size, sex, height, history of hypertension, weight, body mass index (BMI), platelet count, B-type natriuretic peptide (BNP), urea, AST, LAA morphology, AF classification, and antiplatelet therapy.

### 5.3. Model development and validation

**Figure 2.**
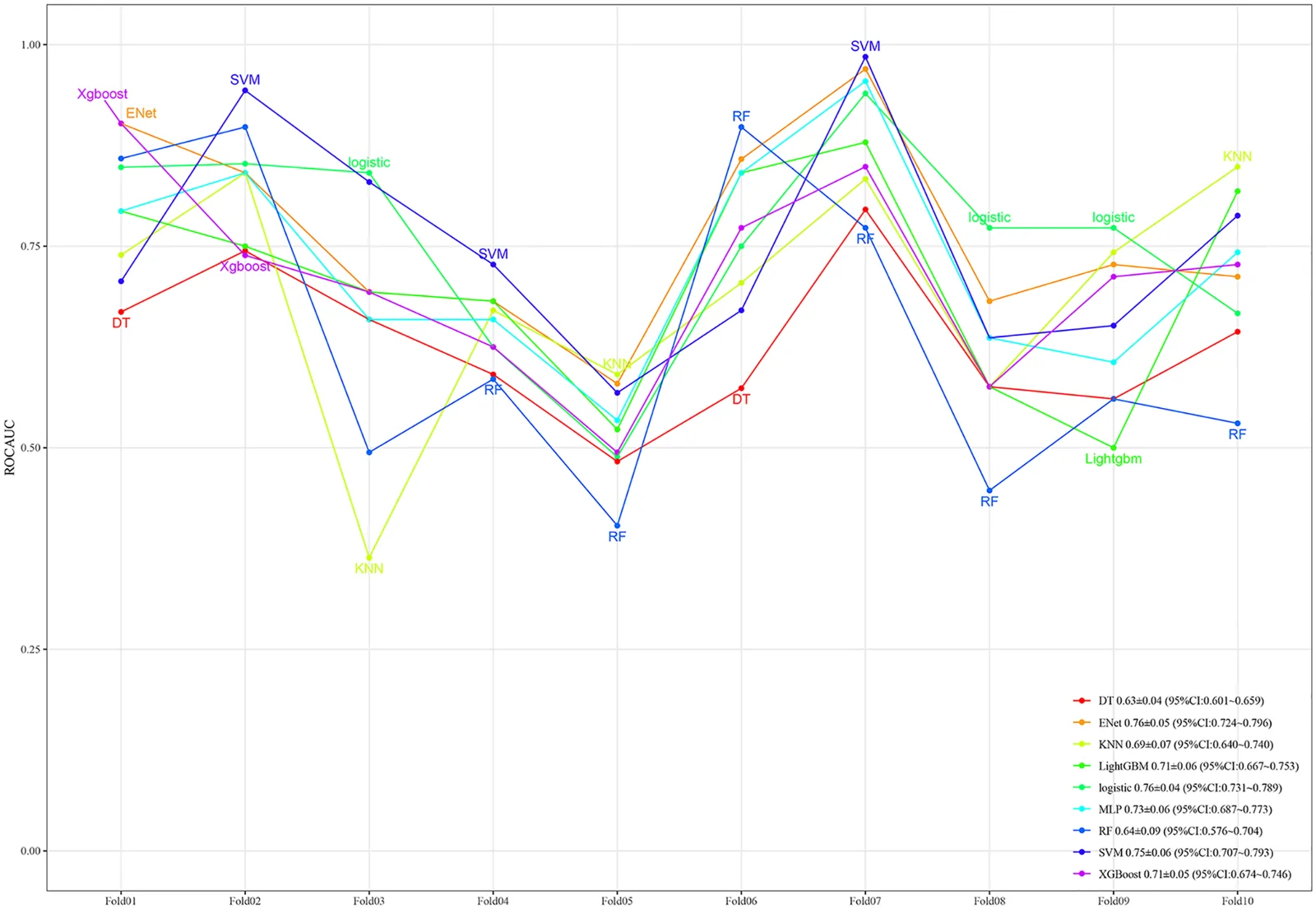
ROC-AUC performance of different machine learning models during ten-fold cross-validation. ROC-AUC = receiver operating characteristic - area under the curve.

We selected 13 variables to construct ML models in the training set. In order to improve model performance on the training set, Bayesian optimization was employed to identify the optimal hyperparameters. To prevent model overfitting, ten-fold CV was employed, and predictive performance was evaluated using the ROC-AUC. The results demonstrated that logistic regression and several ML models achieved favorable ROC-AUC performance: elastic networks, 0.76 ± 0.05 (95% CI: 0.724–0.796); SVM, 0.75 ± 0.06 (95% CI: 0.707–0.793); MLP, 0.73 ± 0.06 (95% CI: 0.687–0.773); light gradient boosting machine, 0.71 ± 0.06 (95% CI: 0.667–0.753); and XGBoost, 0.71 ± 0.05 (95% CI: 0.674–0.746). Detailed model performance is presented in Figure 2.

**Figure 3.**
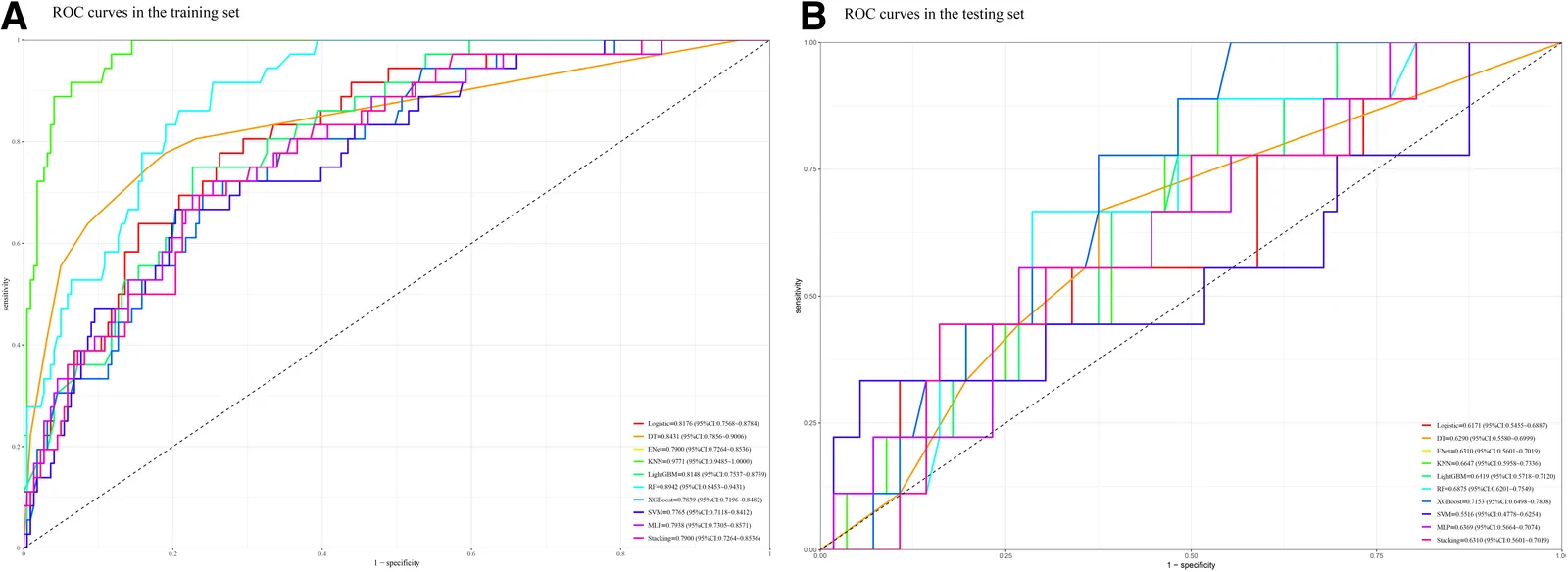
ROC curves of different machine learning models in the training set (A) and testing set (B). ROC = receiver operating characteristic.

Subsequently, model discrimination was further assessed using ROC curve analysis in both the training and testing sets (Fig. 3). In the training set, the KNN model demonstrated favorable discrimination, achieving a ROC-AUC of 0.9771 (95% CI: 0.9485–1.0000), whereas the SVM model showed relatively lower performance (ROC-AUC = 0.7765; 95% CI: 0.7118–0.8412). Further evaluation in the testing set indicated that the XGBoost model exhibited better generalizability, with a ROC-AUC of 0.7153 (95% CI: 0.6498–0.7808). The random forests and KNN models showed comparable but slightly lower performance, with AUC values of 0.6875 (95% CI: 0.6201–0.7549) and 0.6647 (95% CI: 0.5958–0.7336).

**Figure 4.**
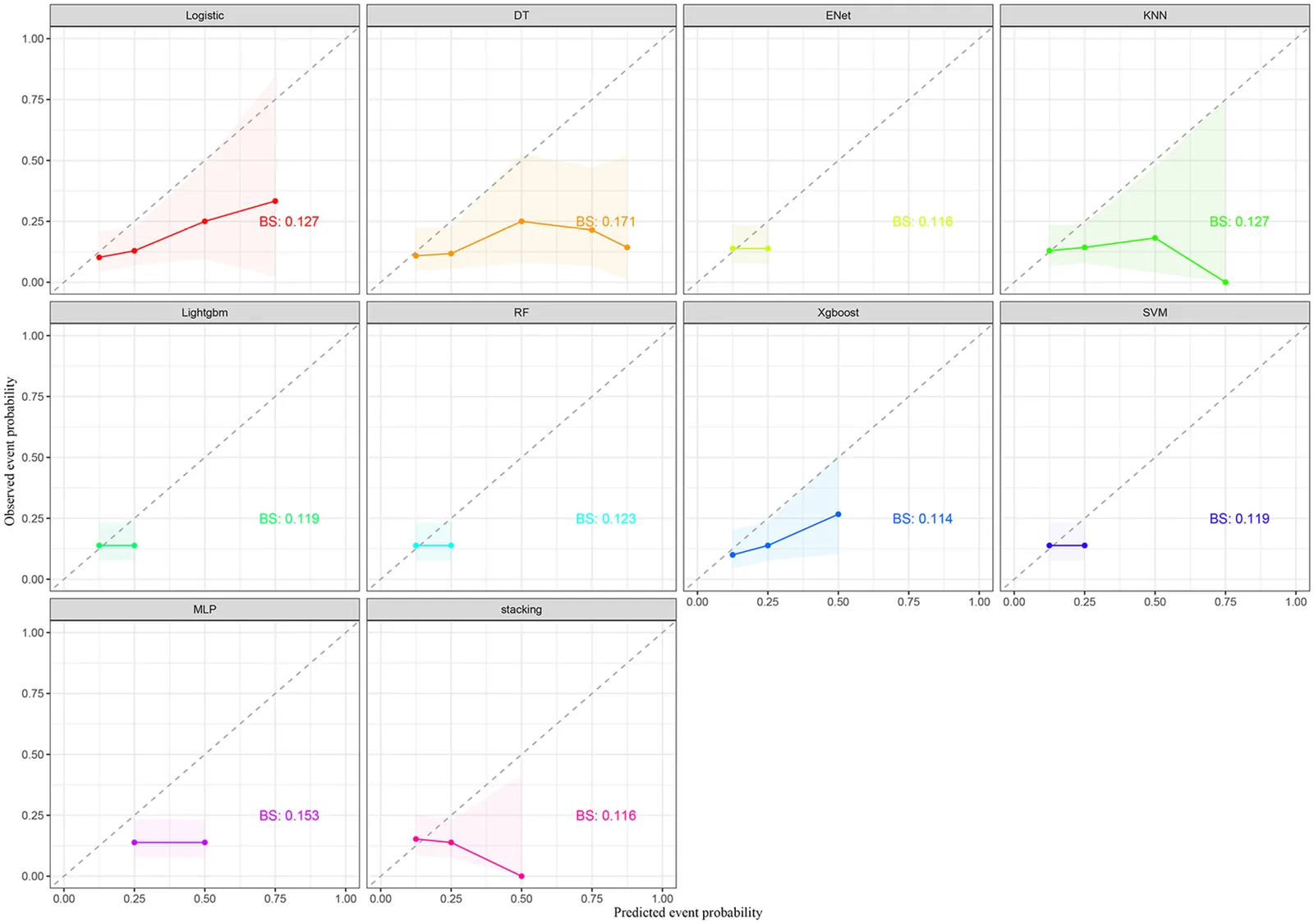
Calibration curves of machine learning models for predicting postoperative arrhythmia.

Calibration curves derived from the testing set indicated that all ML models exhibited acceptable calibration performance (Fig. 4). Among them, the XGBoost model achieved the lowest Brier score (0.114), indicating the best calibration.

**Figure 5.**
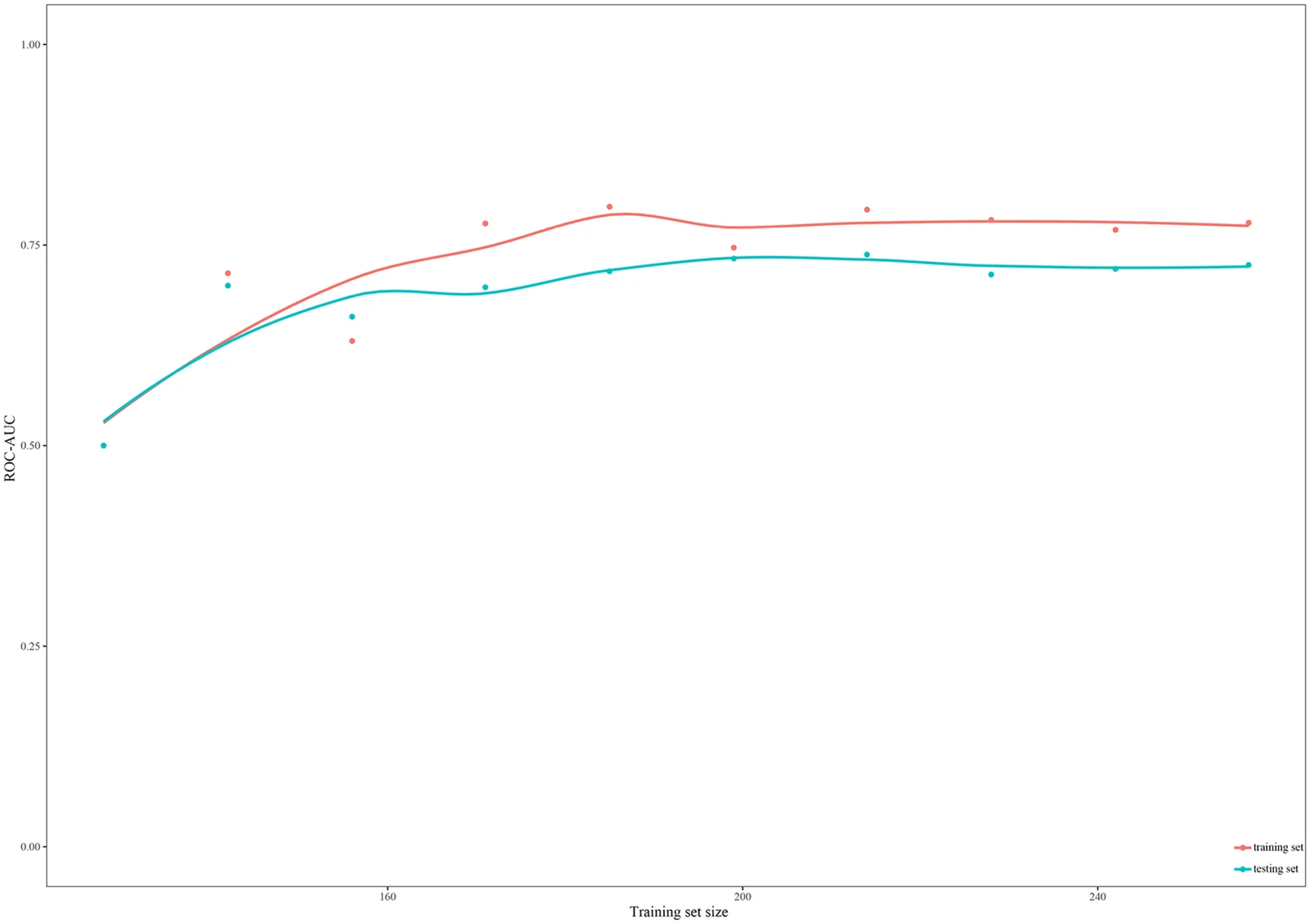
Learning curves of the XGBoost model during training and validation. XGBoost = eXtreme gradient boosting.

To further assess model stability and potential overfitting during training, learning curves of the XGBoost model were examined. Figure 5 shows the learning curves of the model for the training and validation datasets. The training AUC increased and gradually plateaued, while the validation AUC followed a similar trend with slightly lower values, suggesting acceptable generalization performance without evident overfitting.

**Figure 6.**
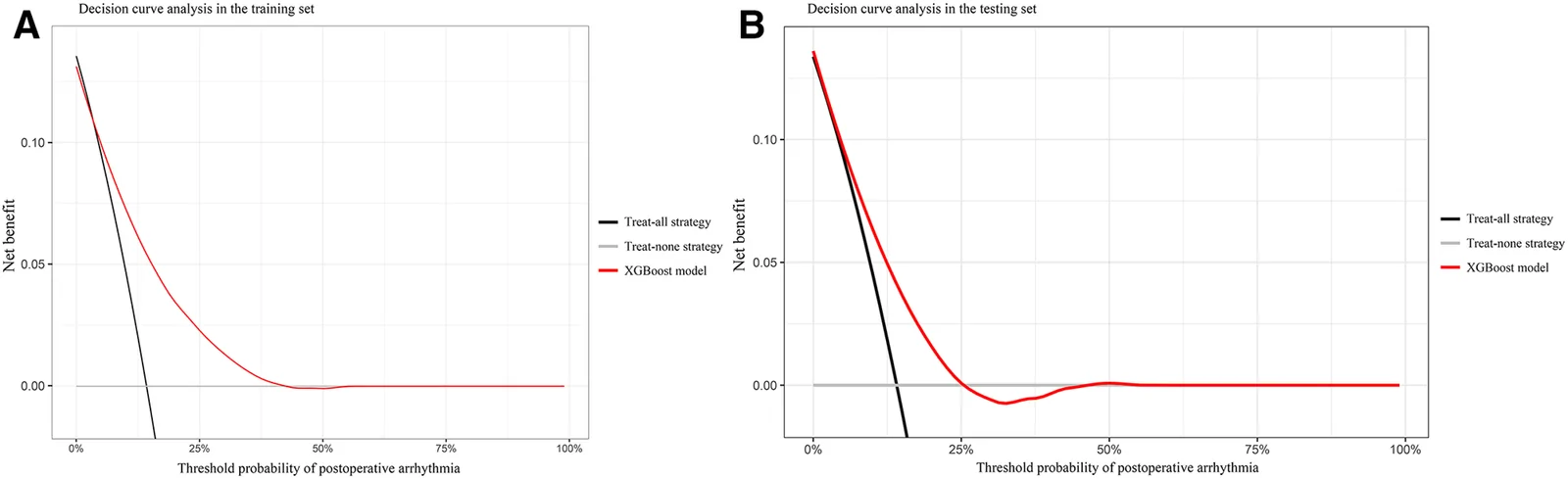
Decision curve analysis of the XGBoost model. Net benefit is plotted against threshold probability in the training set (A) and testing set (B). XGBoost = eXtreme gradient boosting.

DCA was further performed to evaluate the clinical utility of the models. As shown in Figure 6, the x-axis represents the threshold probability of postoperative arrhythmia, and the y-axis represents the net benefit. DCA results from both the training and testing datasets demonstrated that the XGBoost model yielded a clearly defined threshold range of clinical net benefit. In the training set, the model consistently achieved a higher net benefit than the “treat-all” and “treat-none” strategies across threshold probabilities ranging from 5% to 40%. Further validation in the testing set showed that this advantage was stably maintained within a threshold probability range of 0% to 25%. These findings suggest that the XGBoost model may provide reliable support for clinical decision-making within a core effective interval of 0% to 25%, aligning well with the key clinical scenario of screening patients at low to intermediate risk after LAAO and facilitating early preventive strategies for patients at higher risk of postoperative arrhythmia while minimizing unnecessary interventions in low-risk patients.

In addition, the XGBoost model achieved a sensitivity of 0.720 (95% CI: 0.572–0.838), a specificity of 0.691 (95% CI: 0.632–0.744), a positive predictive value of 0.272 (95% CI: 0.197–0.358), and a negative predictive value of 0.939 (95% CI: 0.895–0.967).

Considering the overall performance across the evaluated metrics, the XGBoost model demonstrated the most favorable and well-balanced results.

### 5.4. SHAP analysis of feature importance

To enhance the interpretability of the XGBoost model in predicting postoperative arrhythmia after LAAO, SHAP values were used to quantify the contribution of individual features to model predictions.

**Figure 7.**
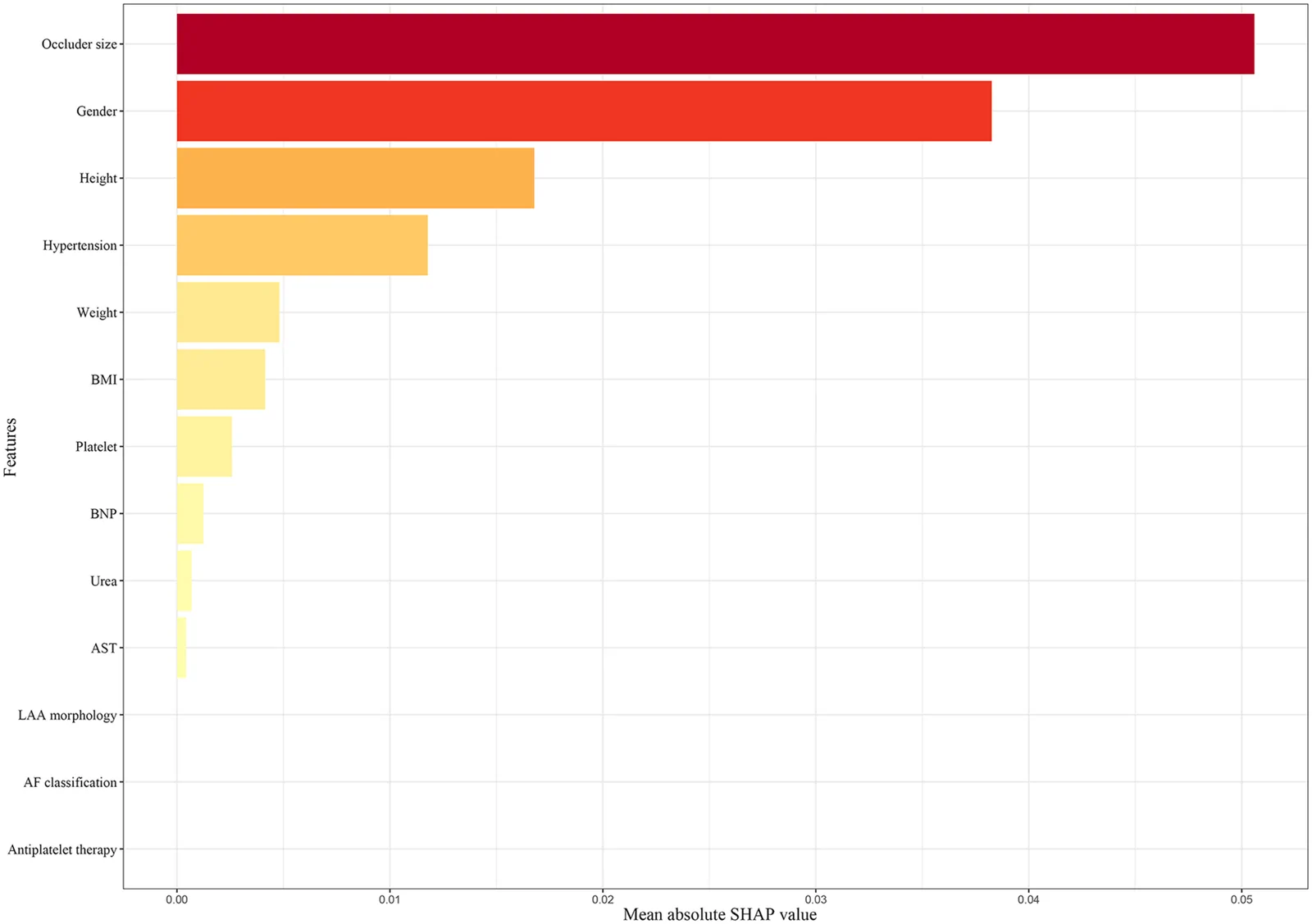
Feature importance of the XGBoost model based on mean SHAP values. XGBoost = eXtreme gradient boosting, SHAP = SHapley Additive exPlanations.

Global feature importance was assessed using the mean absolute SHAP value across the training dataset (Fig. 7). This analysis reflects the overall influence of each variable on the model output, irrespective of direction. Occluder size demonstrated the highest mean absolute SHAP value among all selected features, indicating a prominent contribution to the model’s predictions. Gender, height, and a history of hypertension ranked next in importance, suggesting that these clinical and anthropometric variables also played meaningful roles in risk estimation.

**Figure 8.**
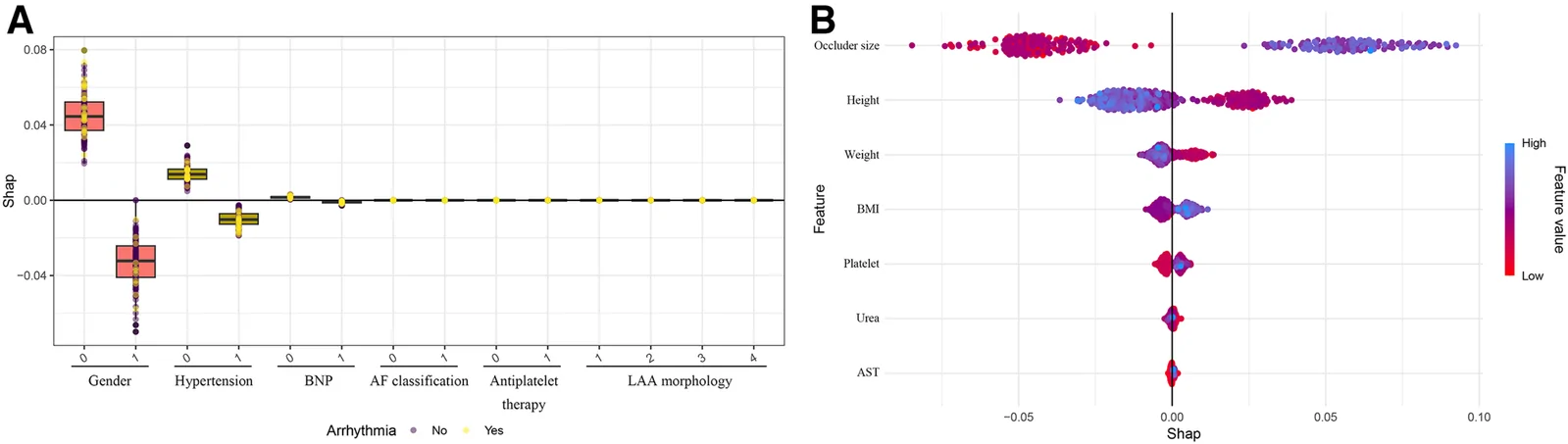
SHAP analysis of predictors in postoperative arrhythmia prediction. (A) SHAP value distributions stratified by postoperative arrhythmia status for categorical variables. (B) SHAP dependence plots for continuous variables. SHAP = SHapley Additive exPlanations.

To further examine how individual features influenced the predicted risk within the model, SHAP dependence plots were generated for continuous variables, whereas SHAP value distributions were compared across outcome groups for categorical variables (Fig. 8). In these analyses, positive SHAP values correspond to an increased predicted probability of postoperative arrhythmia, whereas negative values indicate a decreased predicted probability. For categorical variables, the distribution of SHAP values differed between patients with and without postoperative arrhythmia, indicating heterogeneous contributions of these variables to the model predictions across outcome groups. Variables such as gender, history of hypertension, and BNP demonstrated more pronounced separation of SHAP value distributions, consistent with their higher rankings in the global feature importance analysis. For continuous variables, SHAP dependence plots illustrated the relationship between feature values and their corresponding SHAP values. Among these predictors, occluder size exhibited a relatively wider range of SHAP values compared with other variables, consistent with its stronger global contribution to model output. Other continuous variables, including height, weight, BMI, platelet count, urea, and AST, showed narrower SHAP value distributions, suggesting more modest effects on the predicted risk.

**Figure 9.**
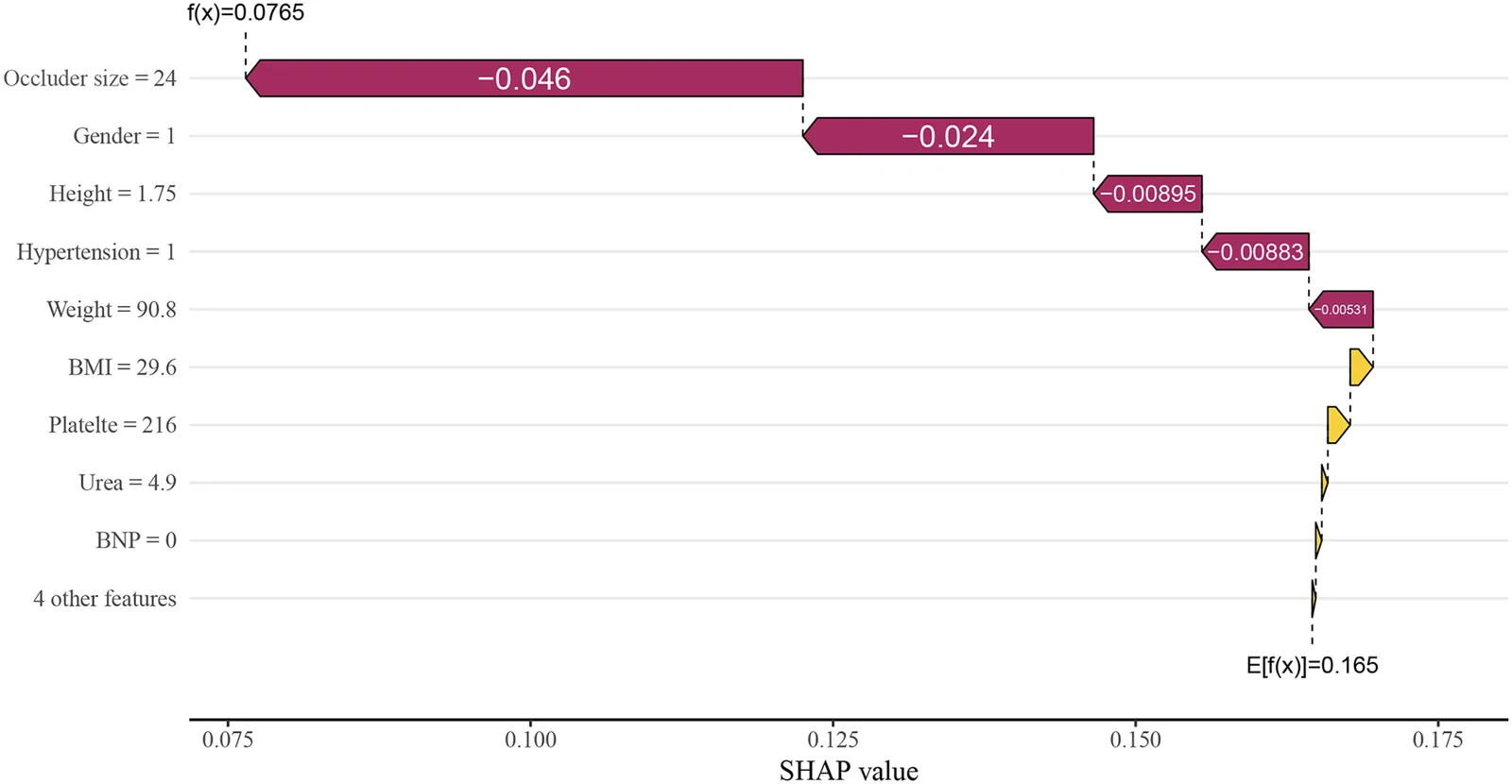
SHAP waterfall plot illustrating individual-level prediction of postoperative arrhythmia. SHAP = SHapley Additive exPlanations.

Individual-level explanations were explored using a SHAP waterfall plot for a representative patient (Fig. 9), which decomposes the model prediction into feature-specific contributions relative to the population baseline. The baseline predicted probability of postoperative arrhythmia was E[f(x)] = 0.165, whereas the final predicted probability for the selected patient was f(x) = 0.0765, reflecting a lower-than-average estimated risk. This reduction was primarily attributable to negative SHAP contributions from several features, with occluder size showing the largest contribution in this case. Other variables exhibited smaller or negligible effects on the individual prediction.

Overall, the SHAP analysis provides transparent global and patient-specific insights into how the XGBoost model integrates multiple clinical and procedural features to estimate the risk of postoperative arrhythmia following LAAO.

### 5.5. Web‑based calculator

**Figure 10.**
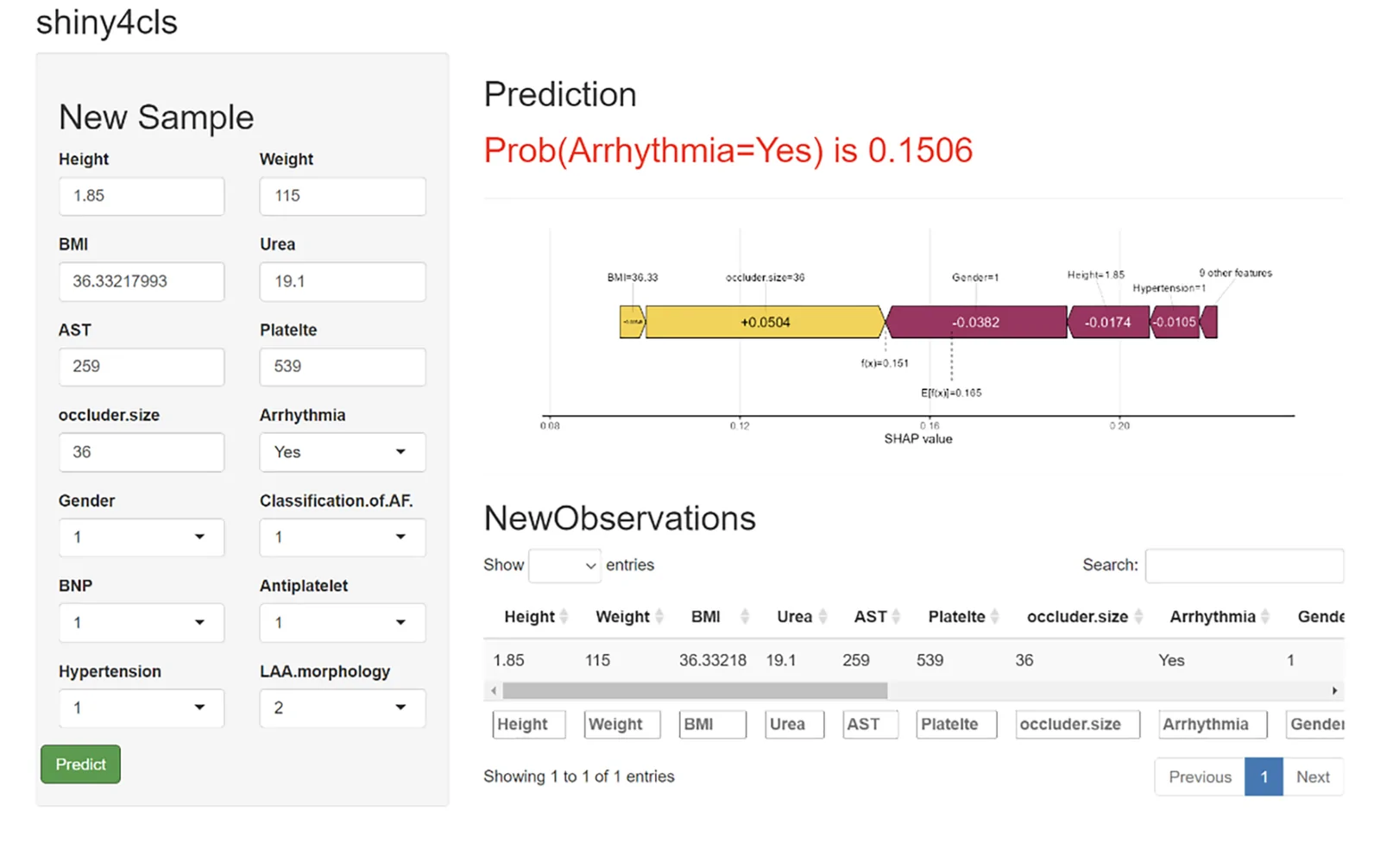
Web-based calculator for individualized prediction of postoperative arrhythmia with SHAP-based interpretability. SHAP = SHapley Additive exPlanations.

A web-based calculator was developed based on the final XGBoost model (Fig. 10). The interface allows clinicians to input patient-specific clinical and procedural variables, after which an individualized probability of postoperative arrhythmia is generated. The calculator also provides a visual display of feature contributions derived from SHAP values, facilitating intuitive understanding of the model’s prediction without implying causal or interventional effects.

## 6. Discussion

Numerous studies have focused on predicting postoperative arrhythmia after LAAO. However, due to patient heterogeneity and the complexity of pathophysiological mechanisms, single assessment indicators or traditional statistical methods are often insufficient for comprehensively integrating the factors that influence postoperative outcomes.^[14]^ ML, with the ability to handle complex datasets and uncover potential relationships between variables, provides an effective approach for developing clinical prediction models.^[15]^

This study employed the SA feature selection algorithm to identify 13 key variables from 35 candidate variables. Eight ML models were constructed, and after Bayesian optimization and ten-fold CV, the XGBoost model was identified as the optimal predictive model. Compared with the findings of previous studies, differences were observed in the reported risk factors. In regression analyses, one study identified older age, BMI, diabetes, previous heart failure, and reduced estimated glomerular filtration rate as significant risk factors for early mortality following LAAO.^[16]^ Another study highlighted the importance of a history of heart failure, left ventricular mass index, E/e’, and abnormal left atrial strain as key factors in predicting postoperative heart failure.^[17]^ Even among studies using ML algorithms, the selection of key risk factors varied. In the realm of ML-based prediction of postoperative AF after cardiac surgery, multiple studies reported that age, gender, left atrial diameter, glomerular filtration rate, and duration of mechanical ventilation were major risk factors for postoperative AF.^[18]^ In contrast, a study focusing on adverse events after LAAO suggested that hypertension, age ≥75 years, diabetes, unstable international normalized ratio, and both CHADS2 and HAS-BLED scores were significant predictors of both short- and long-term adverse events.^[19]^ In our study, SHAP analysis indicated that occluder size, gender, height, and a history of hypertension were key determinants of postoperative arrhythmia. These discrepancies may stem from regional and ethnic differences, variations in baseline patient characteristics, differences in included variables, and divergences in outcome definitions and algorithm choices, further illustrating the complexity and heterogeneity in predicting postoperative arrhythmia after LAAO.

Among the key predictors identified in our SHAP analysis, occluder size emerges as a crucial parameter in LAAO, known to be associated with risks such as device leaks and embolization.^[20,21]^ However, its relationship with postoperative arrhythmia has not been well established. Our study’s SHAP analysis suggests that occluder size could be a key factor in the occurrence of postoperative arrhythmia. We hypothesize that improper fitting of the occluder to the anatomical structure of the LAA could alter the landing zone,^[22]^ and that the mechanical stress and local inflammatory responses at the device’s anchoring points might further affect local myocardial stress,^[23]^ which would potentially trigger electrical derangement. At the cellular level, acute mechanical stretch activates stretch-activated ion channels (SACs), which alters the action potential duration and increases the spatial dispersion of atrial refractoriness.^[24]^ This electrophysiological heterogeneity, combined with the localized acute inflammatory response triggered by the device’s anchoring hooks, creates an ideal substrate for micro-reentrant circuits.

Furthermore, previously identified risk factors for POAF (e.g., gender and history of hypertension) were also found to be important factors influencing postoperative arrhythmia in this study.^[25]^ Differences in the structure of the LAA between genders, and myocardial remodeling induced by hypertension, may contribute to these associations. Additionally, height, which was identified as a significant factor in our study, has not been previously linked to arrhythmia after LAAO. However, an earlier study suggested an association between height and recurrence of AF after catheter ablation in patients with paroxysmal AF.^[26]^ Taller individuals typically possess larger baseline left atrial volumes and surface areas. According to the critical mass hypothesis of AF, a larger atrial surface area provides more physical space for multiple wandering reentrant wavelets to propagate simultaneously without extinguishing themselves.^[27]^ Thus, anthropometric dimensions (such as height) and hypertensive remodeling likely interact cumulatively to enlarge the contiguous electrical mass of the atrium, thereby lowering the threshold for post-procedural tachyarrhythmias,^[28]^ suggesting that height, potentially synergizing with gender-related anatomical differences, may influence left atrial anatomy and contribute to the risk of postoperative arrhythmia.

Complementing these pathophysiological insights, the XGBoost model developed in this study demonstrated acceptable discriminative performance, with an AUC of 0.7153 on the test set. Its Brier score was 0.114, the lowest among all models, indicating that its predicted probabilities aligned well with the actual event probabilities and suggesting reasonable calibration ability. DCA showed that, at threshold probabilities below 0.25, the model provided clinical net benefit, implying that it may assist in clinical decision-making within a specific risk range. Notably, a large-scale cohort study in the United States also found that the XGBoost algorithm outperformed other ML methods in predicting adverse events in LAAO patients, reporting a validation AUC of 0.648 to 0.653.^[29]^ More broadly, ML models in prior literature for predicting perioperative arrhythmias frequently report AUC values ranging from 0.67 to 0.94,^[18]^ placing our model’s performance within an acceptable and pragmatic threshold for clinical risk stratification.

This model and its web-based calculator with personalized risk scores aim to serve as a reference for clinicians designing post-LAAO follow-up protocols. For patients identified as high-risk, clinicians can transition from standard clinical follow-up to proactive preventive strategies. This includes considering extended rhythm monitoring during the critical 2-month post-procedural period. Additionally, these high-risk insights can guide more stringent postoperative blood pressure management and prompt a more cautious regimen of antiarrhythmic drugs (AADs) to suppress early electrical derangement. Conversely, for low-risk patients, unnecessary intensive monitoring can be avoided, minimizing healthcare costs and patient anxiety.

Nevertheless, several limitations must be acknowledged. First, both the training and validation datasets used in this study were sourced from a single-center, and external validation in a geographically or ethnically distinct population was not performed, which limits the generalizability of the model. Future studies should aim to validate the model across multiple centers with broader patient populations. Second, the study only considered baseline characteristics and perioperative static data, without accounting for postoperative medications, dynamic laboratory biomarkers, or environmental factors, which may further influence prediction accuracy.^[30]^ Additionally, the retrospective design inherently relies on existing electronic medical records, which introduces potential documentation biases and limits our ability to capture highly dynamic, post-discharge clinical variables. Third, a formal sample size calculation was not conducted because this study was designed as a single-center exploratory analysis, and the sample size included all eligible patients available during the retrospective study period. Although this approach reflects real-world clinical data, the limited sample size may affect the stability and generalizability of the prediction model. Moreover, only 45 patients developed documented postoperative arrhythmia out of a total cohort of 322 in this study. This limited number of events restricts the capacity of the model to differentiate between specific subtypes of arrhythmias and may affect the statistical stability of certain hyperparameter optimizations. Fourth, the lack of a standardized postoperative rhythm surveillance protocol remains a potential limitation. Although ambulatory ECG monitoring was conducted for 24 hours or longer based on standard clinical practice, the absence of uniform, continuous rhythm monitoring for all patients means that some asymptomatic or brief paroxysmal arrhythmias may have been underdetected. As a result, the predictive performance of the model may not be fully optimized.

In conclusion, this study developed and validated an XGBoost-based ML model for predicting postoperative arrhythmia following LAAO. The model integrated key risk factors, including occluder size, gender, height, and hypertension history, and demonstrated acceptable calibration and discrimination performance. This predictive model offers a valuable tool for risk stratification and clinical management of postoperative arrhythmia. Future research should expand the sample size, conduct multicenter external validation, and incorporate additional dynamic biomarkers and postoperative factors. Moreover, future studies should include populations from diverse geographic regions and ethnicities to further optimize the model’s performance, thereby enhancing its clinical applicability and generalizability.

## Acknowledgments

Part of the data analysis of this study was assisted by Xi’an Evidence Based Pharmaceutical Technology Co., Ltd, and analyzed by Professor Xiong Yang.

## Author contributions

**Data curation:** Liang Xu, Siquan Niu, Shaohua Yang, Yan Wang, Menglu Liu, Xutan Qin.

**Investigation:** Liang Xu, Siquan Niu, Shaohua Yang, Yan Wang, Menglu Liu, Xutan Qin.

**Conceptualization:** Yujie Zhao.

**Funding acquisition:** Yujie Zhao.
